# The Proteome and Lipidome of *Thermococcus kodakarensis* across the Stationary Phase

**DOI:** 10.1155/2016/5938289

**Published:** 2016-05-04

**Authors:** Emma J. Gagen, Marcos Y. Yoshinaga, Franka Garcia Prado, Kai-Uwe Hinrichs, Michael Thomm

**Affiliations:** ^1^Department of Microbiology, University of Regensburg, 93053 Regensburg, Germany; ^2^MARUM-Center for Marine Environmental Sciences and Department of Geosciences, University of Bremen, 28334 Bremen, Germany; ^3^TOPLAB GmbH, Fraunhoferstrasse 18a, 82152 Martinsried, Germany

## Abstract

The majority of cells in nature probably exist in a stationary-phase-like state, due to nutrient limitation in most environments. Studies on bacteria and yeast reveal morphological and physiological changes throughout the stationary phase, which lead to an increased ability to survive prolonged nutrient limitation. However, there is little information on archaeal stationary phase responses. We investigated protein- and lipid-level changes in* Thermococcus kodakarensis* with extended time in the stationary phase. Adaptations to time in stationary phase included increased proportion of membrane lipids with a tetraether backbone, synthesis of proteins that ensure translational fidelity, specific regulation of ABC transporters (upregulation of some, downregulation of others), and upregulation of proteins involved in coenzyme production. Given that the biological mechanism of tetraether synthesis is unknown, we also considered whether any of the protein-level changes in* T. kodakarensis* might shed light on the production of tetraether lipids across the same period. A putative carbon-nitrogen hydrolase, a TldE (a protease in* Escherichia coli*) homologue, and a membrane bound hydrogenase complex subunit were candidates for possible involvement in tetraether-related reactions, while upregulation of adenosylcobalamin synthesis proteins might lend support to a possible radical mechanism as a trigger for tetraether synthesis.

## 1. Introduction

Microorganisms in culture rapidly divide until either an essential nutrient is completely consumed or an inhibitory waste product accumulates, or both, and the population enters a period of no net growth defined as the stationary phase. During the stationary phase, microbial cells undergo various morphological and physiological changes that lead to increased resistance to stress and an ability to survive prolonged nutrient limitation [1]. In nature, the majority of microbial cells probably exist in a stationary phase state due to nutrient deprivation in most environments [2]. Cellular processes occurring beyond the onset of stationary phase have been investigated in bacteria (for reviews, see [2, 3]) and in yeast (e.g., see [4]), but there is a dearth of information for Archaea. A literature search revealed only that Dinger et al. [5] investigated archaeal histones during late stationary phase of* Thermococcus zilligii* and Adams et al. [6] noted 28 genes (despite not revealing their identity) that were upregulated in* Pyrococcus furiosus* in the late stationary phase compared to the start of stationary phase.

During our studies on the membrane lipids of Archaea, we incidentally noticed that lipid profiles changed as cultures progressed beyond the onset of stationary phase. Intriguingly, the proportion of membrane lipids comprised of a tetraether backbone frequently seemed to increase with extended time in stationary phase and we speculated that this may be an archaeal adaptation in response to stationary phase or to nutrient limiting conditions. Tetraether lipids are membrane spanning and their presence in archaeal membranes is thought to minimise metabolic stress and futile ion cycling [7].

In the present study, we investigated archaeal stationary phase responses using a proteomics and lipidomics approach in a model archaeon,* Thermococcus kodakarensis*. The aim of the study was to (1) understand the mechanisms that Archaea employ to cope with survival during stationary phase and (2) examine whether any of these processes may relate to the observed increased tetraether production throughout the stationary phase. Production of diether lipids in Archaea is relatively well understood; however, the mechanism of formation of tetraether lipids is poorly constrained and may involve novel biochemistry (e.g., for a review, see [8]).

## 2. Materials and Methods

### 2.1. Growth of* T. kodakarensis*

*T. kodakarensis* was grown as previously described [52] with yeast extract, tryptone, and pyruvate as carbon and energy sources, in a 15 L fermentor at 85°C except without flushing of the reactor headspace. One litre of culture was harvested at the beginning of stationary phase and twelve hours later. Cells from 500 mL culture at both time points were analysed for lipids as outlined below and cells from the remaining 500 mL of culture at both time points were sent for comparative proteomic analysis at TOPLAB (Martinsried, Germany) by isotope-coded label protein. Cells were stored at −80°C until the respective analyses.

### 2.2. Lipid Analysis

Lipids were extracted according to Sturt et al. [53] with slight modifications. In brief, samples were lyophilized and weighed. Dry cell mass (0.09–0.14 g) was combined with precombusted sea sand (2 g) and extracted four times. Samples were extracted by ultrasonication into a solvent mixture (v : v) of methanol (MeOH), dichloromethane (DCM), and aqueous buffer (2 : 1 : 0.8). A phosphate buffer (8.7 g L^−1^ KH_2_PO_4_, pH 7.4) was used for the first two steps, and a trichloroacetic acid buffer (50 g L^−1^, pH 2) was used for the final two steps. Supernatants were pooled in a separation funnel and DCM and water were added in order to allow optimal phase separation. After transferring the organic phase, the aqueous phase was extracted three more times with DCM. Pooled organic layers were then washed three times with deionized MilliQ water. The final extract was gently evaporated under N_2_ flow and stored at −20°C.

Chromatographic separation was achieved on a Waters Acquity UPLC Amide column and a Waters Acquity BEH C_18_ column, in normal and reverse phase for, respectively, polar and core lipids [54]. High-performance liquid chromatography (HPLC, Dionex Ultimate 3000RS UHPLC) was coupled to a Bruker maXis quadrupole time-of-flight mass spectrometer (Q ToF-MS, Bruker Daltonics, Bremen, Germany) equipped with an electrospray ion source (ESI). Detection of lipids was performed in positive ionization mode while scanning a mass-to-charge (*m*/*z*) range from 150 to 2000. For each mass spectrum (MS) full scan, MS/MS experiments were obtained in data-dependent mode, targeting the most abundant ions. Active exclusion was used to limit the fragmentation of a given ion (3 times every 0.5 min) and thus allowed us to also obtain MS/MS data of less abundant ions. Lipid identification was achieved by monitoring exact masses of possible parent ions (present as either H^+^ or NH_4_^+^ adducts) in combination with characteristic fragmentation patterns as outlined by Yoshinaga et al. [55] and supported by compound identities revealed in previous studies [52, 56]. Lipid quantification was obtained by comparison of both core and polar lipids of* T. kodakarensis* relative to the peak area of the internal standard 1,2-dihenarachidoyl-sn-glycero-3-phosphocholine (C_21_-PC, Avanti Lipids). As no authentic standards are available for all compounds, concentrations are reported based on response factors of commercially available or purified standards relative to known amounts of the internal standard C_21_-PC. To account for the response factors, we performed calibration curves relative to C_21_-PC using four concentrations of the commercial and purified standards (0.5, 1, 5, and 10 ng), before and after measuring the samples from* T. kodakarensis*. We used phosphatidylethanolamine archaeol (PE-AR, Avanti Polar Lipids Inc., USA) for quantification of diether phospholipids and phosphatidylglycerol-monoglycosyl-glycerol-di-biphytanyl-glycerol-tetraether, PG-GDGT-G (from Matreya LLC, Pleasant Gap, PA, USA), for the tetraether phospholipids. Core lipids of* T. kodakarensis* were quantified using core AR and core GDGT isolated from* Archaeoglobus fulgidus*, as described by Zhu et al. [57]. Ionization of standards PE-AR, PG-GDGT-G, and core AR and core GDGT was lower than C_21_-PC and the following response factors were applied: 1.8, 9.8, 1.8, and 4.2, respectively.

### 2.3. Isotope-Coded Label Protein (ICPL) Sample Preparation

Isotope-coded protein label (ICPL*™*) is a top-down proteomics approach, which is based on differential isotopic labelling of proteins derived from different cell states with either light or heavy tags directed to all lysine residues and protein N-termini [58]. After labelling, the samples are combined, cleaved into peptides, and analysed using LC-MS/MS. Since isotopes have identical physicochemical properties, the light labelled peptides coelute with their heavy counterparts and are simultaneously analysed in the mass spectrometer. Quantitative analysis is then performed by comparing the relative signal intensities of the light and heavy labelled peptide in the MS-spectra. Finally, the peptides are identified by MS/MS analysis followed by protein database searching. For each protein, the quantified peptide pairs are summarised and the mean of their ratio is reported [59, 60].

Cell pellets for protein analysis were lysed on ice in 200 *μ*L ICPL lysis buffer (Serva Electrophoresis GmbH, Heidelberg, Germany) and homogenized by grinding using a grinding kit (GE Healthcare, catalogue number 80-6483-37) before centrifugation at 12,000 ×g for 10 min at 4°C to remove cell debris. The supernatants were transferred to new tubes and after protein concentration determination by Bradford assay [61] (Bio-Rad Protein Assay Dye Reagent Concentrate #500-0006), total protein was adjusted to 5 mg mL^−1^ for ICPL labelling using the lysis buffer. For both samples, 100 *μ*g of protein was labelled using the ICPL*™* Quadruplex Plus kit (Serva Electrophoresis GmbH). Briefly, the N-terminus and the amino groups of lysine were labelled as per the manufacturer's instructions with the nicotinoyloxysuccinimide reagents ICPL_0 and ICPL_6 (^13^C_6_) (Serva Electrophoresis GmbH) for the sample collected at the beginning of stationary phase and with ICPL_4 (^2^H_4_) and ICPL_10 (^2^H_4_^13^C_6_) (Serva Electrophoresis GmbH) for the sample collected twelve hours later. A pool of both samples served as material for the reference run. The pooled samples were aliquoted and labelled separately with all four ICPL labels. Subsequent analysis steps were performed analogously to those of the analytical run. Multiplets of the reference run showed equal intensity due to the labelling of the same material and contained all detectable proteins due to the combining of both samples. Knowledge of their retention time and masses enabled the recognition of highly or on-off regulated proteins in the analytical MS run of the samples [62]. Enzymatic cleavage was performed using sequencing grade trypsin (porcine, Serva Electrophoresis GmbH) and MS_grade Glu-C (Protea Biosciences Inc.). Peptides were acidified using 1% trifluoroacetic acid.

### 2.4. Mass Spectrometric Analysis of* T. kodakarensis* Proteins

For nanoscale liquid chromatography coupled to electrospray ionization-tandem mass spectrometry (nLC-ESI-MS/MS), 0.685 *μ*g of protein digest was injected. Peptides were separated using an analytical column (reversed phase C18, 50 cm, 60°C) with a 140 min linear gradient (A: 0.1% formic acid, B: 80% acetonitrile and 0.1% formic acid) at a flow rate of 250 nL min^−1^ with the gradient from 5 to 50% of solution B. Mass spectrometry was performed on a linear ion trap mass spectrometer (LTQ Orbitrap Elite, Thermo Scientific) operating in positive ion mode online coupled to the nano-LC. MS consisted of a cycle combining one full MS scan (mass range: 300–1500 *m*/*z*) with ten data-dependent MS/MS events (CID: 35% collision energy).

### 2.5. Proteomic Analysis and Database Queries

Raw data were converted to mzXML format using the Trans-Proteomic Pipeline [63]. Peak detection, deconvolution, deisotoping, and quantification were done using ICPL-ESIQuant [62]. Quadruplet detection was initially performed for the sample run and reference run separately and quadruplets detected in the reference run were used to search for incomplete quadruplets in the sample run. Mascot identification data were imported with a threshold ion score of 19 where individual ion scores > 19 indicated identity or extensive homology (*p* < 0.05). The database used was constructed from the published genome of* T. kodakarensis* available from GenBank (accession number AP006878) [64]. Moreover, all sequences were randomized and added to the target sequences giving a concatenated database of 4612 sequences. The decoy sequences were used to calculate the false discovery rate (FDR). Four separate database queries were performed, always using one of the four ICPL labels as a fixed modification. Proteins with fewer than two quadruplets and/or a coefficient of variation above 40% were excluded from further analysis. The FDR of the identified peptides was calculated by the formula FDR = 2*∗*FP/(FP + TP), where FP is the number of false positives and TP is the number of true positives [65]. 2027 true positive hits and 61 false positive hits resulted in an FDR of 6.0%.

### 2.6. *T. kodakarensis* Proteins Upregulated after Twelve Hours in Stationary Phase

To determine which proteins had been upregulated between the start of stationary phase (ICPL labels 0 and 6) and twelve hours later (ICPL labels 4 and 10), protein expression ratios were compared for the four ICPL labels (4 : 0, 4 : 6, 10 : 0, and 10 : 6). Proteins that were at least twofold upregulated or twofold downregulated as indicated by at least two of the ratios were considered to be of interest.

### 2.7. Bioinformatic Analysis Upregulated Proteins

Proteins that were up- or downregulated twelve hours after the onset of stationary phase were assessed bioinformatically using the information from the protein annotation at the NCBI, the detected conserved domains in the proteins [66], BLAST homology to other proteins [67, 68], information at BRENDA [69], and literature searches.

### 2.8. Assessment of Upregulated Proteins for Possible Involvement in Tetraether-Related Reactions

To consider whether any of the proteins upregulated in* T. kodakarensis* across the stationary phase might have characteristics that could make them candidates for direct involvement in tetraether formation from diether precursors, the upregulated proteins were assessed against various criteria.

(1) The presence of possible transmembrane helices in proteins was investigated using three different methods: searches for hidden Markov models with TMHMM [70], statistical comparison of naturally occurring transmembrane proteins using TMbase [71], and assignment based on preference functions using the SPLIT server [72].

(2) Protein identity to squalene epoxidases was determined by BLAST analysis against protein P32476 from* Saccharomyces cerevisiae* and protein Q75W20 from* Panax ginseng*. Homology to a squalene epoxidase was considered because terbinafine, a squalene epoxidase inhibitor, also inhibits tetraether lipid formation [73]; therefore, potentially, the enzyme(s) catalysing tetraether formation share some similarities with squalene epoxidases. Homology was considered significant at an alignment *E*-value of less than 1*e* ^−3^.

(3) The presence of genes encoding similar proteins in other Archaea was investigated by BLAST searches restricted (using the organism name function) to each of the following phylogenetically, physiologically, and ecologically diverse Archaea that have all been reported to produce tetraethers:* Aeropyrum pernix*,* Acidilobus saccharovorans*,* Archaeoglobus fulgidus*,* Caldisphaera lagunensis*,* Fervidicoccus fontis*,* Ignicoccus hospitalis*,* Methanocaldococcus jannaschii*,* Methanopyrus kandleri*,* Methanospirillum hungatei*,* Methanothermobacter thermautotrophicus*,* Nitrosopumilus maritimus*,* Pyrolobus fumarii*,* Sulfolobus solfataricus*, and* Thermoplasma acidophilum*. Specific BLAST searches were also made against the genomes of* Halobacterium halobium* and* Methanosarcina acetivorans*, which have not been reported to produce tetraethers, as well as the genome of* Nanoarchaeum equitans*. A protein involved in tetraether formation might be conserved amongst the majority of Archaea, as other lipid synthesis enzymes are; however, it was expected to be absent from the genome of* N. equitans. N. equitans* has one of the smallest archaeal genomes known to date; it does not encode any genes for proteins involved in lipid synthesis [74] but instead derives its membrane lipids from its host* I. hospitalis* [75] and we therefore expected* N. equitans* to be unlikely to have retained genes for proteins involved directly in tetraether lipid synthesis.

(4) Proteins with a predicted oxidoreductase function were considered of interest as tetraether synthesis by a mechanism related to condensation of two diether lipids would occur by oxidoreduction.

(5) Proteins with an annotation associated with lipids, prenyl groups, or isoprenoids or with genomic context neighbouring an enzyme annotated as such were also considered of interest.

## 3. Results and Discussion

### 3.1. *T. kodakarensis* Lipids throughout the Stationary Phase

**Figure 1:**
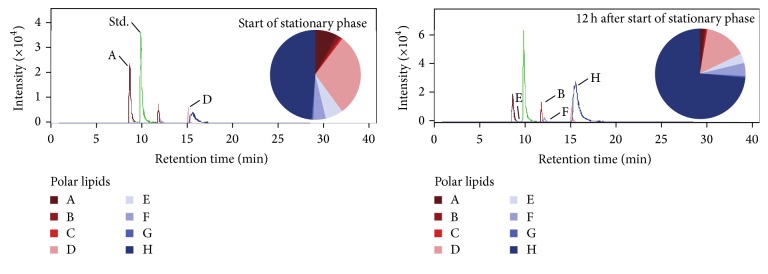
Selected ion chromatogram of major polar lipids of* T. kodakarensis* during HPLC-ESI-MS analysis, showing compound intensity and elution time at the beginning of stationary phase and twelve hours later. Note that polar lipids are shown along with the internal standard (Std.) for comparison. Pie charts display the relative abundance of diether (in red) and tetraether (in blue) polar lipids of* T. kodakarensis*. Diethers: A = phosphatidylglycerol; B = phosphatidyl-N-acetylhexosamine; C = C_5_H_8_ + phosphatidylinositolhexosamine; D = phosphatidylinositol. Tetraethers: E = phosphatidylglycerol; F = phosphatidyl-N-acetylhexosamine; G = phosphatidic acid; H = phosphatidylinositol.

In this study, tetraethers represented the majority of total intact polar lipids in* T. kodakarensis*, in agreement with previous investigations in Thermococcales [56, 76], however contrasting with the higher proportions of diether than tetraether polar lipids reported by Meador et al. [52]. Potentially, this discrepancy may stem from analytical biases (i.e., extraction protocols and HPLC-MS methods, e.g., Cario et al. [77]) and/or slightly different growth conditions of* T. kodakarensis* between the two studies, with flushing of bioreactor headspace [52] not undertaken in the present study, making it more like a batch culture system. This factor may also explain the somewhat lower cellular lipid concentrations observed in this study compared to our previous study by Meador et al. [52]. Nevertheless, the headgroup composition of polar lipids was similar to that reported by Meador et al. [52], with both diethers and tetraethers being mainly phospholipids, with phosphatidylinositol and phosphatidylglycerol as major headgroups (Figure 1). Across the twelve hours in stationary phase, there was a 3.6-fold and 1.2-fold increase in the concentrations of tetraether and diether polar lipids, respectively (Table 1). Except for phosphatidylglycerol diether, increased concentrations of tetraether and diether polar lipids were reflected in all headgroups (Figure 1). When considering total core lipids (i.e., the polar headgroup-free glycerolipid backbone), a 2.2-fold increase in tetraethers and a 1.4-fold decrease in diethers were observed (Table 1).* T. kodakarensis* is known to regulate its membrane lipid composition in response to growth stage and environmental factors [52, 56] and our findings extend that observation to reveal that changes in lipid composition in* T. kodakarensis* continue beyond the active growth stages and throughout culture stationary phase. Our results showing an increase in tetraether lipids across this period are in line with the idea suggested by Valentine et al. [7] that tetraether lipids serve to minimise loss of ions across the membrane and thus reduce metabolic stress, which in this case would aid survival throughout prolonged stationary phase conditions.

### 3.2. Upregulated Proteins in* T. kodakarensis* Cells Twelve Hours after the Onset of Stationary Phase

Of the 2,306 predicted proteins encoded by the genome of* T. kodakarensis*, 657 were identified in our samples and of these 336 contained at least one ICPL labelled peptide and were thus able to be quantified between the start of stationary phase and 12 hours later. Comparing the relevant ICPL label ratios (Table 2), 32 proteins were found to be at least twofold upregulated by at least two ICPL ratios (31 when considering only the average of the relevant ICPL ratios) and 12 proteins were on average at least 2-fold downregulated twelve hours after the onset of stationary phase. At a peptide identity significance threshold of *p* < 0.05, the false discovery rate (FDR) using this approach was estimated to be 6.0%.

### 3.3. Functional Categorisation of Upregulated Proteins

#### 3.3.1. Transcription and Translation

Approximately half of the upregulated proteins in* T. kodakarensis* across stationary phase were implicated in transcription, translation, and RNA/DNA synthesis and repair (Table 2). Upregulation of posttranscriptional and posttranslational modifiers and proteins involved in correcting misacylated tRNAs indicates that* T. kodakarensis* directs energy into ensuring translational fidelity as the stationary phase progresses. This is in contrast with observations in bacterial models where genes encoding transcription and translation are downregulated in response to growth arresting conditions [78] and reduced translational fidelity has been observed when bacterial cells enter stasis or start to experience carbon starvation [3, 79]. However, proteins involved in transcription, translation, and DNA repair have been listed in the general stress response proteome for all three domains of life [80].

A broad range transcriptional regulator, TrmB (transcriptional regulator of mal operon), was also upregulated during the stationary phase, confirming that gene expression in* T. kodakarensis* is regulated across this period, likely in response to nutrient limited conditions. TrmB was first characterized as a maltose and trehalose responsive repressor of transcription of the genes encoding sugar ABC transporters [23] and has since been shown to act on up to 113 archaeal promoters to either activate or repress transcription of diverse genes in response to nutritional starvation [24].

#### 3.3.2. Central Metabolism and Energy Generation

As expected, proteins involved in uptake of amino acids and sugars were upregulated throughout the stationary phase, probably in response to nutritional stress (e.g., [26]) as amino acids are a carbon source for* T. kodakarensis* [81]. However, regulation of these proteins seems to be very specific as four other proteins with ABC domains, predicted to play a role in transport of peptides and other molecules, were significantly downregulated across the stationary phase (Table 2) and one of these (YP_184217) was an ABC transporter that Jia et al. [34] previously found to be upregulated under oxidative stress conditions. Thus, expression of various transport systems in* T. kodakarensis* seems to be tightly controlled, probably in response to specific carbon requirements, rather than as a general cellular stress response. Many of the ABC transport systems in* T. kodakarensis* are uncharacterised (for both substrate and directionality) and would benefit from further research. Upregulation of a peptidase (YP_183697) may also have been a nutritional response in* T. kodakarensis*, although in* E. coli* peptidases are upregulated under starvation conditions for the purpose of continued de novo protein synthesis [3, 82] which could also be the case for* T. kodakarensis*.

It seems likely that fermentative growth would have been slowing as the* T. kodakarensis* culture continued through stationary phase; therefore, upregulation of a protein annotated as NADH-quinone oxidoreductase subunit (YP_184506) of the membrane bound hydrogenase complex (mbh) was surprising (Table 2). A second oxidoreductase, YP_183806, thought to play a role in glycerol catabolism in heterotrophic Archaea (and not involved with glycerophosphate backbone of archaeal lipids which have different glycerol stereochemistry) [30] was also upregulated across the stationary phase, despite the absence of added glycerol to the culture. The gene encoding the homologous enzyme in* Escherichia coli* (ygaF) is reportedly induced by carbon starvation and at stationary phase [31] and the enzyme belongs to a broad group of oxidoreductases and thus may act on substrates other than glycerol.

Upregulation of ornithine carbamoyltransferase with time in stationary phase was also a surprising observation in* T. kodakarensis* as ornithine carbamoyltransferases typically catalyse the formation of citrulline from carbamoyl phosphate and ornithine, during arginine biosynthesis. However,* T. kodakarensis* is an arginine auxotroph [32]; therefore, this protein is clearly not functioning for the purpose of arginine biosynthesis and must play another role in this organism. Legrain et al. [33] suggested that it functions in reverse to convert citrulline into ornithine and carbamoyl-P as a source of energy. While its specific function remains ambiguous, potentially ornithine carbamoyltransferase is important in stress responses in* T. kodakarensis* as Jia et al. [34] found the same protein to be upregulated in in response to heat and oxidative stress.

#### 3.3.3. Coenzyme Production

Enzymes central to the production of coenzyme A and vitamin B12 (adenosylcobalamin) were upregulated in* T. kodakarensis* throughout the stationary phase (YP_184227, YP_183265, Table 2). Upregulation of other proteins (e.g., YP_183167, Table 2) indicated that processes requiring coenzyme A were occurring during the stationary phase in* T. kodakarensis*. Kültz [80] has also noted that upregulation of gene products involved in coenzyme A metabolism is a stress response conserved amongst the three domains of life. Adenosylcobalamin is the most complex coenzyme known and it assists enzymes by the provision of radicals, enabling catalysis of unusual isomerization or methylation reactions if radicals are also provided from S-adenosylmethionine (SAM) [36, 83, 84]. Upregulation of another protein involved in the adenosylcobalamin production pathway has been observed for* T. kodakarensis* under salt stress (YP_183265, Jia et al. [34]) so, potentially, this vitamin also plays a role in various stress responses in* T. kodakarensis*.

Upregulation of a predicted methylthioribose-1-phosphate isomerase (YP_182969) during the stationary phase would suggest the functioning of the methionine salvage pathway and thus regeneration of SAM in* T. kodakarensis* across the stationary phase. SAM is a key molecule in a wide range of biochemical processes [39, 40, 85] including diphthine synthesis and posttranscriptional modifications such as formation of N(1)-methyladenine or N(1)-methylguanine at position 9 of tRNA, for example, tRNA^Ala^ and tRNA^Asp^ [9], which were processes that seem to have been upregulated during the stationary phase of* T. kodakarensis* (Table 2). However, Sato et al. [41] have previously shown that* T. kodakarensis* does not possess a functional methionine salvage pathway* in vivo*; thus, upregulation of the predicted methylthioribose-1-phosphate isomerase across the stationary phase is not for the purpose of SAM regeneration. The enzyme belongs to a family of proteins that are homologous to eukaryotic translation initiation factor 2B (eIF2B), involved in GTP recycling in eukaryotes [86]. Sato et al. [41] found that a homologue of an eIF2B subunit in* T. kodakarensis* actually demonstrates ribulose-1,5-bisphosphate synthase activity (while YP_182969 did not show this activity). Thus, YP_182969 is likely to also have an as-yet-unknown biological function* in vivo* and would benefit from detailed biochemical characterisation.

#### 3.3.4. Lipid Synthesis

Upregulation of HMG-CoA reductase (YP_183327) indicated that active synthesis of isoprenoids was occurring in* T. kodakarensis* with extended time in stationary phase. The cellular need for new isoprenoids may have been for the purpose of protein prenylation, in response to stress conditions (e.g., [87]). Upregulation of HMG-CoA reductase has been reported for a halotolerant archaeon* Haloferax volcanii* [88] as well as halotolerant fungi [87] in response to nonoptimal growth (salt) conditions previously. Alternatively, the need for isoprenoids may have been for the synthesis of new lipids in* T. kodakarensis* which would be consistent with our observation that total IPLs increased with time in stationary phase (Table 1).

#### 3.3.5. Hypothetical Proteins and Unknown Functions

Six proteins of unknown function or general prediction only were also upregulated in* T. kodakarensis* with time in stationary phase and it is difficult to speculate on their possible function aside from broad predictions based on other members of their superfamily. Amongst them was a putative carbon-nitrogen hydrolase, a metal-dependent phosphohydrolase that has also been observed to be upregulated in* T. kodakarensis* in response to salt stress [34] and a TldE (a protease in* Escherichia coli*) homologue (Table 2).

### 3.4. Proteins Potentially Involved in Production of Tetraether Lipids throughout the Stationary Phase

Given that both the proteome and the lipidome of* T. kodakarensis* changed across the twelve hours in stationary phase, we took the opportunity to investigate whether any of the upregulated proteins across this period might shed light on the production of tetraether lipids, across this same period. To date, research that has been done to determine the mechanism of tetraether synthesis in the Archaea has been* in vivo* using labelled precursor and substrates and/or terbinafine as an inhibitor of tetraether formation. The results of these studies are at times conflicting [73, 89–93] and both the structure of the precursors and the mechanism of condensation remain unresolved. A recent hypothesis [94], which is unsupported by direct experimental observations, is that condensation of the two phytanyl chains occurs in a 1′ to 4′ fashion before the chains are attached to glycerol moieties. Villanueva et al. [94] proposed phytoene synthase as a possible enzyme to catalyse this condensation; however, the absence of phytoene synthase in most archaeal genomes means that this hypothesis fails to explain tetraether synthesis in the majority of Archaea, including* T. kodakarensis*.

Screening criteria to consider whether proteins upregulated in the present study (i.e., concomitant with an increase in tetraether production) could be candidates for involvement in tetraether formation included distribution of the protein in the Archaea, protein similarity to squalene epoxidases, annotation associated with lipids, and the presence of transmembrane domains (see Materials and Methods for explanatory notes on the criteria used, Table 3 for results). Of the proteins classified as being associated with lipids, YP_184329 (annotated as a class 9 apolipoprotein N-acyltransferase) may interact with lipids in the manner that other class 9 acyltransferases interact in, transferring the 1-carbonyl of a phospholipid to the amino group of a lipoprotein precursor (for mechanism, see [95, 96]). However, despite its annotation, YP_184329 was more similar to class 13 nitrilases than to class 9 nitrilases and the substrates of this class are unknown [42]. YP_184329 was predicted to have transmembrane helices by all three methods tested (Table 3) and has homologues in all but two of the archaeal genomes screened (and a homologue is lacking in the genome of* N. equitans* as expected as well) and therefore may be a candidate for involvement in tetraether-related reactions potentially by a mechanism reminiscent of a nitrilase.

A TldE homologue (YP_182912) currently annotated as a zinc-dependent protease was one of only two of the upregulated proteins in this study that demonstrated any significant alignment similarity to a squalene epoxidase (Table 3). Also, interestingly, TldD and TldE (two proteins that form a proteolytic complex possibly involved in modulation of gyrase function in* Escherichia coli* [47]) are widely distributed in the Archaea though they are missing in the two archaeal orders not reported to produce tetraethers (Nanoarchaeota and Haloarchaeota) [45]. Furthermore, YP_182912 was predicted to contain a transmembrane helix (Table 3), suggesting that it could interact with membrane lipids. Both YP_184329 and YP_182912 would benefit from further research to determine their function* in vivo* and the possibility of involvement in tetraether-related reactions.

The two upregulated proteins with predicted oxidoreductase activity were of interest, because the mechanism by which two diethers might condense to form a tetraether would conceivably be by an oxidation-reduction reaction. NADH-quinone oxidoreductase (YP_184506) in the mbh complex shows homology to subunits of complex I which catalyse the transfer of electrons from, for example, NADH to quinone molecules in a reaction associated with proton translocation across the membrane [97]. As quinones have not been detected in Thermococcales species [98] and* T. kodakarensis* employs electron bifurcation to generate an electrochemical potential for ATP synthesis, YP_184506 probably transfers electrons from ferredoxin to H^+^ [99] rather than to quinones. However, this protein is an interesting candidate for some sort of involvement in the condensation of two diether isoprenoids, given its extensive homology to complex I proteins [100] that interact with isoprenoid molecules in the membrane, transfer electrons, and effect oxidoreductase activity. Furthermore, a homologue of this protein is distributed in all archaeal genomes except* N. equitans*. The second oxidoreductase (YP_183806) upregulated concomitantly with tetraether formation also had some of the characteristics that make it of interest as a potential player in tetraether formation, including predicted transmembrane domains and alignment similarity to a squalene epoxidase (Table 3). However, its limited distribution (Table 3) means that if it plays a role in tetraether formation, it is not by a mechanism conserved amongst all Archaea.

An additional observation was the upregulation of an adenosylcobalamin synthesis protein, as well as enzymes catalysing reactions requiring SAM, indicating that radical-assisted reactions were occurring in* T. kodakarensis* during the stationary phase. There are a variety of cellular processes and unusual reactions that rely on the methyl group or radicals donated from SAM or from adenosylcobalamin or both [83, 84]. With respect to tetraether formation, a radical reaction involving cobalamin has already been proposed [92, 101] as a mechanism that could explain tetraether synthesis in a manner similar to the one proposed for formation of diabolic acid in bacteria by head-to-head condensation of two fatty acids [102]. That hypothesis is worth revisiting in light of our observation of upregulation of proteins involved in adenosylcobalamin synthesis concomitant with increased tetraether formation.

## 4. Conclusions

Across the stationary phase, we observed protein-level and lipid-level changes in* Thermococcus kodakarensis* that shed light on archaeal adaptations in response to extended time in the stationary phase and/or to nutrient stress. These adaptations included increased proportion of lipids with a tetraether backbone in the membrane, an investment in synthesis of proteins that ensure translational fidelity, very specific regulation of ABC transporters (upregulation of some and downregulation of others), and upregulation of proteins involved in coenzyme production as well as upregulation of various proteins whose cellular role remains ambiguous or completely unknown. Two of these proteins, whose function remains ambiguous, were also found to be upregulated under other nonoptimal conditions in a previous study on* T. kodakarensis* [34] and thus could be part of a general stress response in* T. kodakarensis*.

As the mechanism of tetraether lipid synthesis remains unresolved and the enzyme most recently proposed as responsible for it (phytoene synthase [94]) is notably absent in* T. kodakarensis* and most other archaeal genomes, we investigated whether any of the proteins upregulated in the present study (concomitant with an increase in tetraether production) could be candidates for involvement in tetraether production. A putative carbon-nitrogen hydrolase (YP_184329), a TldE homologue (YP_182912), and a subunit of the mbh complex (YP184506) were the most likely candidates from the list of proteins upregulated with time in stationary phase and each would benefit from further investigation. Upregulation of adenosylcobalamin synthesis proteins concomitant with tetraether synthesis would lend support to the possibility that a radical mechanism is the trigger for tetraether synthesis [101] and may also be worth considering in the future.

## Figures and Tables

**Table 1:** Di- and tetraether core and intact polar lipids (IPLs) of *T. kodakarensis* at the beginning of stationary phase and twelve hours later.

	Start of stationary phase^a^	Twelve hours after start of stationary phase^a^	Fold change
IPL diethers	0.05	0.06	+1.2
IPL tetraethers	0.08	0.29	+3.6
Total IPLs	0.13	0.35	+2.7
Core lipid diethers	0.19	0.13	−1.4
Core lipid tetraethers	0.02	0.04	+2.2
Total core lipids	0.20	0.13	−1.2

^a^Lipids are expressed as fg of lipid per cell.

**Table 2:** Proteins of *T. kodakarensis* that were up- or downregulated at least twofold^a^ between the start of stationary phase and twelve hours later, grouped by broad functional categories.

Genome accession	Annotated name	Average fold change	Broad level protein function prediction	Notes
YP_182835	tRNA (guanine-N2)-dimethyltransferase	**4.20 ± 0.63**	Posttranscriptional modification	Catalyses the SAM-dependent formation of N(1)-methyladenine or N(1)-methylguanine at position 9 in tRNA, which may contribute to thermostability of archaeal tRNAs [9]
YP_183167	tRNA(Met) cytidine acetyltransferase	**4.84 ± 2.20**	Posttranscriptional modification	Catalyses acetyl-CoA-dependent N4-acetylation of tRNA^Met^ important for recognition of the AUG codon and translational fidelity [10]
YP_184653	Lysyl-tRNA synthetase	**4.31 ± 0.30**	tRNA formation	Catalyses formation of lysyl-tRNA
YP_183321	Glutamyl-tRNA(Gln) amidotransferase subunit D	**3.57 ± 0.30**	tRNA editing	Part of a complex that catalyses transamidation to form Gln-tRNA^Gln^ from misacylated Glu-tRNA^Gln^ [11]
YP_183397	Alanyl-tRNA editing protein AlaX	**1.81 ± 0.39**	tRNA editing	Catalyses hydrolysis of misacylated tRNA^Ala^ [12]
YP_183917	30S ribosomal protein S11	**3.41 ± 0.63**	Translation	Part of the 30S subunit of the ribosome, the molecular machinery for protein biosynthesis [13]
YP_183938	50S ribosomal protein L6	**4.88 ± 2.30**	Translation	Part of the 50S subunit of the ribosome, the molecular machinery for protein biosynthesis [13]
YP_183954	50S ribosomal protein L4P	**2.03 ± 0.51**	Translation	Part of the 50S subunit of the ribosome, the molecular machinery for protein biosynthesis [13]
YP_182519	Diphthine synthase	**4.75 ± 0.27**	Translation	Catalyses the SAM-dependent trimethylation of an intermediate in diphthamide formation from histidine [14]; diphthamide is required for archaeal translation elongation factor 2 [15]
YP_184539	Protein kinase	**5.12 ± 0.38**	Posttranslational modification	Component of the KEOPS complex responsible for formation of N6-threonylcarbamoyladenosine, important for translational fidelity [16, 17]
YP_182619	Hypothetical protein TK0206	**3.69 ± 0.48**	RNA/DNA replication and repair	A predicted RAD55 domain comprises half the protein; RAD55 has been implicated in DNA repair and signal transduction [18]
YP_182979	RNA helicase	**4.59 ± 0.34**	RNA/DNA replication and repair	Belongs to DEAD-like helicase superfamily, involved in ATP-dependent RNA or DNA unwinding [19]
YP_183694	Endonuclease	**3.69 ± 0.31**	RNA/DNA replication and repair	5′-flap endonuclease and 5′-3′-exonuclease activity, characterised in *Pyrococcus horikoshii* [20]
YP_183841	Hypothetical protein TK1428	**2.54 ± 0.39**	RNA/DNA replication and repair	Cleavage and polyadenylation specificity factor subunit-like protein; these are predicted in Archaea to be RNases [21]
YP_184316	DNA polymerase II large subunit	**4.84 ± 2.20**	DNA replication and repair	Catalytic subunit of DNA polymerase, genome replication [22]
YP_184182	Transcriptional regulator	**3.63 ± 0.32**	Transcription regulation	TrmB is a transcriptional regulator first characterized as a repressor of transcription of genes encoding sugar ABC transporters [23] and later shown in *Halobacterium salinarum* to act on up to 113 archaeal promoters in response to nutritional stress [24]
YP_183072	Ribose ABC transporter permease	**4.59 ± 0.34**	Amino acid cycling and energy generation	ABC transport domain suggests involvement in amino acid/sugar uptake, although ABC transporters may be channels or exporters or serve a regulatory function [25]
YP_184170	Peptide ABC transporter ATPase	**4.31 ± 0.30**	Amino acid cycling and energy generation	ABC transport domain suggests involvement in amino acid/sugar uptake, although ABC transporters may be channels or exporters or serve a regulatory function [25]
YP_183697	Peptidase	**4.21 ± 0.34**	Amino acid cycling and energy generation	Intracellular protease with a type 1 glutamine amidotransferase domain, homologous to proteins thought to hydrolyze small peptides for nutrition [26] and upregulated under peptide-limiting conditions [27] in other Thermococcales
YP_184506	NADH-quinone oxidoreductase	**4.01 ± 0.64**	Energy generation	Subunit of the membrane bound hydrogenase (mbh) complex, involved in disposal of excess reducing equivalents, essential in fermentative growth of *T. kodakarensis* [28, 29]
YP_183806	Glycerol 3-phosphate dehydrogenase	**4.90 ± 0.42**	Energy generation	Involved in glycerol catabolism in heterotrophic Archaea [30]; it belongs to protein superfamily L-2-hydroxyglutarate oxidase; gene encoding homologous enzyme in *Escherichia coli* (ygaF) is induced by carbon starvation and stationary phase [31]
YP_183284	Ornithine carbamoyltransferase	**3.36 ± 0.38**	Amino acid biosynthesis (?)	Predicted to play a role in arginine biosynthesis via ornithine; however, *T. kodakarensis* is an arginine auxotroph [32]; therefore, the role of this enzyme is unclear, potentially functioning in reverse to convert citrulline to ornithine [33]; it may be a stress response factor [34]
YP_184227	L-Tyrosine decarboxylase	**4.19 ± 1.85**	Coenzyme production	Catalyses formation of beta-alanine for coenzyme A production [35]
YP_183265	Hypothetical protein TK0853	**2.59 ± 0.46**	Coenzyme production	Shows strong homology to nicotinate-nucleotide-dimethylbenzimidazole (NaMN:DMB) phosphoribosyl transferase, involved in formation of alpha-ribazole-5′-phosphate, a precursor of adenosylcobalamin (vitamin B12) [36, 37]
YP_183327	3-Hydroxy-3-methylglutaryl-CoA reductase	**4.59 ± 0.34**	Lipid synthesis	Catalyses the rate-limiting step in isoprenoid biosynthesis (formation of mevalonate from 3-hydroxy-3-methylglutaryl-CoA) [38]
YP_182969	Methylthioribose-1-phosphate isomerase	**2.91 ± 0.57**	Function unknown	Predicted to play a role in the methionine salvage pathway [39, 40]; however, *T. kodakarensis* lacks a function methionine salvage pathway [41]; therefore, the role of this enzyme is unknown
YP_184329	Apolipoprotein N-acyltransferase	**4.31 ± 0.30**	Function unknown	Shows strong identity to protein Ph0642 (accession 1J31) within class 13 of the nitrilase superfamily, therefore potentially a carbon-nitrogen hydrolase [42, 43]
YP_182427	Oxetanocin	**8.93 ± 4.22**	Function unknown	Belongs to superfamily of metal-dependent phosphohydrolases whose function is unknown [44]; it may be a stress response protein [34]
YP_182912	Zinc-dependent protease	**3.64 ± 0.58**	Function unknown	Identity to a TldE homologue (Sso0661) that does not display protease activity [45]; TldE homologues may play a role in modulation of DNA gyrase [46] or antibiotic secretion [47]
YP_183662	Hypothetical protein TK1249	**2.13 ± 0.53**	Function unknown	Shows identity to proteins classified as hypothetical proteins within either aconitase or DUF521 superfamilies
YP_183924	Hypothetical protein TK1511	**3.25 ± 0.52**	Function unknown	Belongs to uncharacterized protein family UPF0150
YP_184398	Hypothetical membrane protein	**5.66 ± 0.49**	Function unknown	Thermococcales-specific hypothetical protein with no conserved domains, potentially membrane associated
YP_184630	2-Amino-3-ketobutyrate coenzyme A ligase	−2.11 ± 0.32	Amino acid cycling	Involved in conversion of threonine to glycine [48]
YP_184213	Oligopeptide ABC transporter ATP-binding protein	−2.50 ± 0.21	Amino acid cycling	ABC transport domain suggests involvement in amino acid/sugar uptake, although ABC transporters may be channels or exporters or serve a regulatory function [25]
YP_183708	Predicted thiol protease	−48.88 ± 1.13	Protein turnover	Belongs to C1 peptidase family of endo- and exopeptidases
YP_183338	Phenylalanyl-tRNA ligase subunit beta	−2.32 ± 0.08	Translation	Catalyses attachment of phenylalanine to its cognate tRNA [49]
YP_183718	Probable translation initiation factor IF-2	−8.65 ± 1.04	Translation	Archaeal/eukaryotic translation initiation factor 5B, homologous to prokaryotic initiation factor 2 which promotes binding of the initiator tRNA to the ribosome during translation; the predicted protein sequence contains an intein that is posttranslationally excised
YP_183212	DNA topoisomerase VI subunit B	−2.31 ± 0.08	DNA replication and repair	Part of a type IIB DNA topoisomerase, involved in manipulating the topological state of DNA [50]
YP_182643	ABC-type multidrug transport system, ATPase component	−2.29 ± 0.08	Transport or DNA replication and repair	May be the ATPase component of a system involved in transport of molecules across the membrane or may be an ABC ATPase, involved in DNA repair, translation, or gene regulation [51]
YP_184173	CGP-CTERM sorting domain-containing protein	−4.31 ± 0.36	Transport (?)	Contains the Cys-Gly-Pro motif and C-terminus transmembrane domain found in various Thermococcales proteins, but of unknown function (potentially related to lipid modification); it shows similarity to ABC transporter substrate-binding proteins and thus may be involved in transport of compounds across the membrane
YP_182982	CGP-CTERM sorting domain-containing protein	−2.46 ± 0.24	Function unknown	Hypothetical protein with a putative ABC transport domain and a Cys-Gly-Pro motif followed by a transmembrane domain at the C-terminus; such CGP-CTERM domains have so far only been found in members of the Thermococcales and their function is speculative though they may be related to lipid modification
YP_183717	Hypothetical protein TK1304	−3.92 ± 0.34	Function unknown	Hypothetical protein with no conserved domains detected; it appears to be Thermococcales specific
YP_184217	Peptide ABC transporter substrate-binding protein	−4.88 ± 0.37	Function unknown	ABC transport domain suggests involvement in amino acid/sugar uptake, although ABC transporters may be channels or exporters or serve a regulatory function [25]
YP_183593	Hypothetical protein TK1180	−14.25 ± 0.63	Function unknown	Thermococcalesspecific protein of unknown function

^a^Protein expression ratios were compared for the relevant ICPL labels (ICPL4 : ICPL0, ICPL4 : ICPL6, ICPL10 : ICPL0, and ICPL10 : IPL6) and proteins that were at least twofold upregulated or twofold downregulated as indicated by at least two of the ratios were considered to be of interest. The average of the four ratios and standard error of the mean are presented in the table.

**Table 3:** Screening criteria used to consider whether upregulated proteins might play a role in tetraether lipid formation.

Genome accession	Annotated name	Number of screened archaeal genomes with a homologue present^a^	Homologue present or absent in genome of *N. equitans* ^b^	Demonstrating identity to a squalene epoxidase^c^	Number of predicted transmembrane helices by 3 different methods^d^
YP_182427	Oxetanocin	9	Absent	No	0, 0, 0
YP_182519	Diphthine synthase	15	Present	No	0, 1, 0
YP_182619	Hypothetical protein TK0206	0	Absent	No	0, 1, 0
YP_182835	tRNA (guanine-N2)-dimethyltransferase	7	Present	No	0, 0, 0
YP_182912	Zinc-dependent protease	14	Absent	5*e* ^−4^ to P32476	0, 1, 1
YP_182969	Methylthioribose-1-phosphate isomerase	16	Absent	No	0, 2, 1
YP_182979	RNA helicase	16	Absent	No	0, 1, 0
YP_183072	Ribose ABC transporter permease	4	Absent	No	10, 9, 6
YP_183167	tRNA(Met) cytidine acetyltransferase	10	Present	No	0, 1, 1
YP_183265	Hypothetical protein TK0853	15	Absent	No	0, 2, 1
YP_183284	Ornithine carbamoyltransferase	16	Absent	No	0, 0, 0
YP_183321	Glutamyl-tRNA(Gln) amidotransferase subunit D	16	Present	No	0, 1, 0
YP_183327	3-Hydroxy-3-methylglutaryl-CoA reductase	13	Absent	No	0, 2, 1
YP_183397	Alanyl-tRNA editing protein AlaX	15	Present	No	0, 0, 0
YP_183662	Hypothetical protein TK1249	13	Absent	No	0, 0, 0
YP_183694	Endonuclease	16	Present	No	0, 0, 0
YP_183697	Peptidase	10	Absent	No	0, 0, 0
YP_183806	Glycerol 3-phosphate dehydrogenase	5	Absent	1*e* ^−4^ to Q75W20	0, 2, 1
YP_183841	Hypothetical protein TK1428	16	Present	No	0, 1, 0
YP_183917	30S ribosomal protein S11	16	Present	No	0, 0, 0
YP_183924	Hypothetical protein TK1511	3	Absent	No	0, 0, 0
YP_183938	50S ribosomal protein L6	16	Present	No	0, 0, 0
YP_183954	50S ribosomal protein L4P	16	Present	No	0, 0, 0
YP_184170	Peptide ABC transporter ATPase	16	Absent	No	0, 0, 0
YP_184182	Transcriptional regulator	8	Absent	No	0, 1, 0
YP_184227	L-Tyrosine decarboxylase	9	Absent	No	0, 3, 1
YP_184316	DNA polymerase II large subunit	9	Present	No	0, 2, 0
YP_184329	Apolipoprotein N-acyltransferase	11	Absent	No	1, 2, 1
YP_184398	Hypothetical membrane protein	0	Absent	No	0, 2, 1
YP_184506	NADH-quinone oxidoreductase	16	Absent	No	0, 0, 0
YP_184539	Protein kinase	16	Present	No	0, 0, 0
YP_184653	Lysyl-tRNA synthetase	10	Present	No	0, 0, 0

^a^Sixteen different archaeal genomes were screened (refer to Materials and Methods) and a homologue was considered to be present for proteins with an *E*-value < 1*e* ^−5^ and coverage across > 30% of the protein.

^b^More stringent criteria (*E*-value < 1*e* ^−10^ and coverage across > 60% of the protein) were used for determining the presence of homologues in *N. equitans* in order not to discard any potentially relevant proteins unnecessarily.

^c^The potential level of similarity between a tetraether related enzyme and a squalene epoxidase is completely unknown; therefore, an *E*-value of < 1*e* ^−3^ was considered to be of interest, across any level of coverage in the protein.

^d^Number of transmembrane helices predicted by TMHMM, TMbase, and SPLIT (given in that order in the table). See Materials and Methods for details on transmembrane prediction tools.
